# A Case of Steven-Johnson Syndrome Secondary to Bumetanide

**DOI:** 10.7759/cureus.97038

**Published:** 2025-11-17

**Authors:** Fariyah Waseem, Jainy Parikh

**Affiliations:** 1 Medicine, Lancashire Teaching Hospital National Health Service (NHS) Foundation Trust, Preston, GBR

**Keywords:** bumetanide, diuretic allergy, rash, sjs, steven johnson syndome

## Abstract

Steven-Johnson syndrome (SJS) is a severe cutaneous adverse reaction that can be triggered by medications. Bumetanide, which is a loop diuretic often prescribed for edema associated with heart, liver, and kidney conditions, is a sulfonamide-containing drug that has been linked to severe cutaneous adverse reactions (SCARs). This case report details a confirmed instance of SJS secondary to bumetanide administration in a 64-year-old gentleman. Prompt identification, discontinuation of the offending agent, and supportive care led to gradual recovery.

## Introduction

Stevens-Johnson syndrome (SJS) is a life-threatening dermatologic emergency characterised by extensive epidermal necrosis and mucosal involvement. Drug exposure accounts for more than 80% of cases [1], with high-risk agents including sulfonamides, antiepileptic medications, and allopurinol. Bumetanide, a potent loop diuretic, is only rarely reported as a causative agent [2,3]. Other recognised triggers include infections such as Mycoplasma pneumoniae and herpes simplex virus, as well as malignancy; however, in a subset of patients, no definitive precipitant is identified [4].

The pathophysiology of SJS is believed to represent a type IV hypersensitivity reaction mediated by cytotoxic T lymphocytes and natural killer cells, resulting in widespread keratinocyte apoptosis [5]. Genetic susceptibility plays a contributory role-certain HLA alleles and slow drug-metabolising phenotypes can lead to accumulation of reactive metabolites that act as haptens, triggering immune activation [6].

Clinically, SJS is often preceded by a nonspecific prodrome of fever, malaise, and sore throat, followed by a rapid onset of erythematous macules, targetoid lesions, and mucosal involvement of the eyes, mouth, and genitalia. Within days, epidermal detachment and blistering develop. The extent of skin detachment differentiates SJS (<10% body-surface area) from toxic epidermal necrolysis (TEN >30%) [7].

Early recognition, prompt withdrawal of the offending agent, and comprehensive supportive care-often in an intensive care or burn-unit setting-are essential to improving outcomes [8]. Various immunomodulatory treatments, including corticosteroids, intravenous immunoglobulin (IVIG), and cyclosporine, have been used with variable and sometimes conflicting evidence regarding efficacy [9-11].

## Case presentation

**Figure 1:**
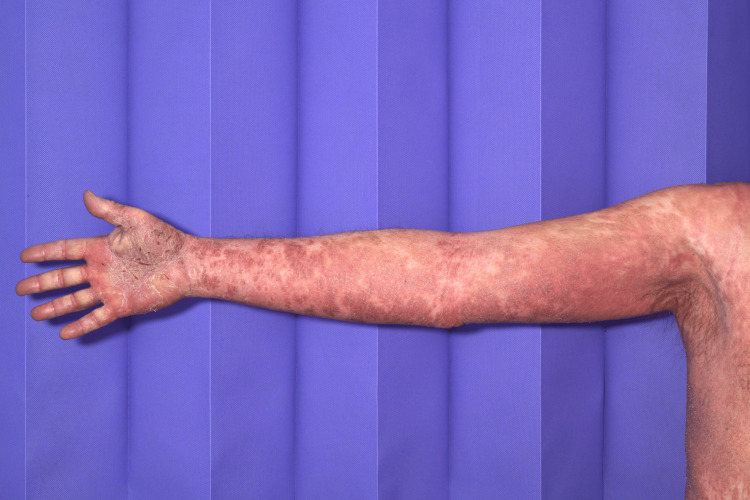
The patient had widespread SJS maculopapular rashes over arm and hands SJS: Steven-Johnson Syndrome.

**Figure 2:**
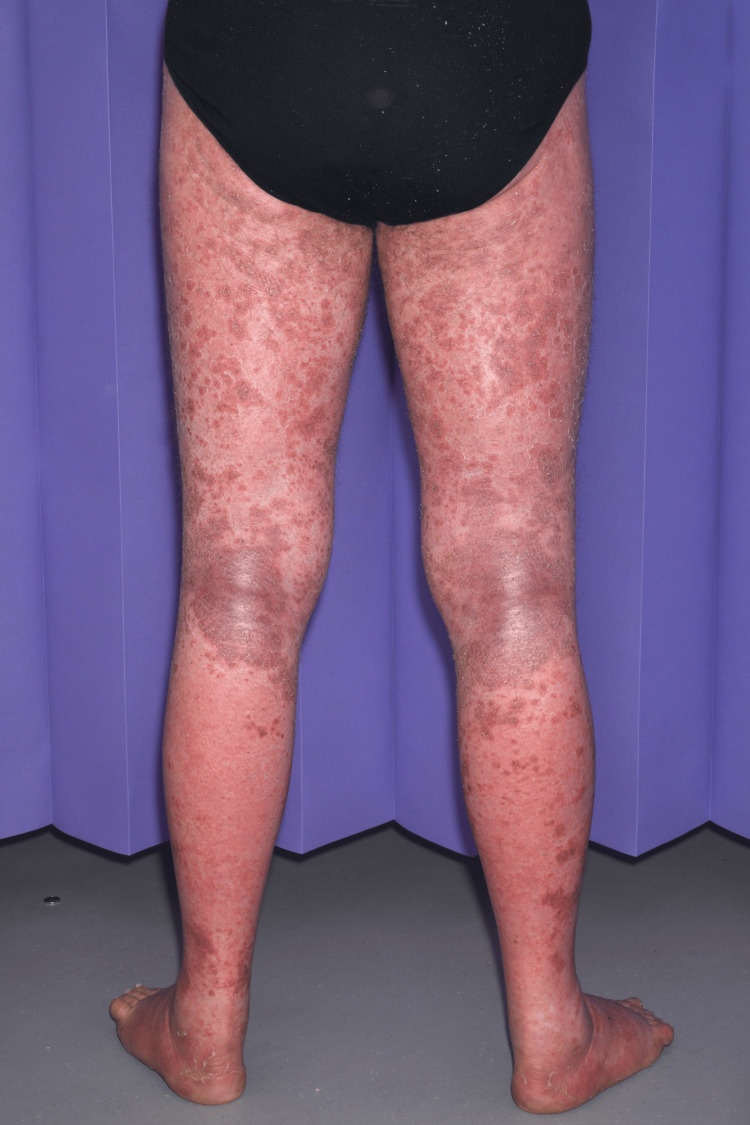
The patient had SJS rashes after bumetanide over legs SJS: Steven-Johnson Syndrome.

**Figure 3:**
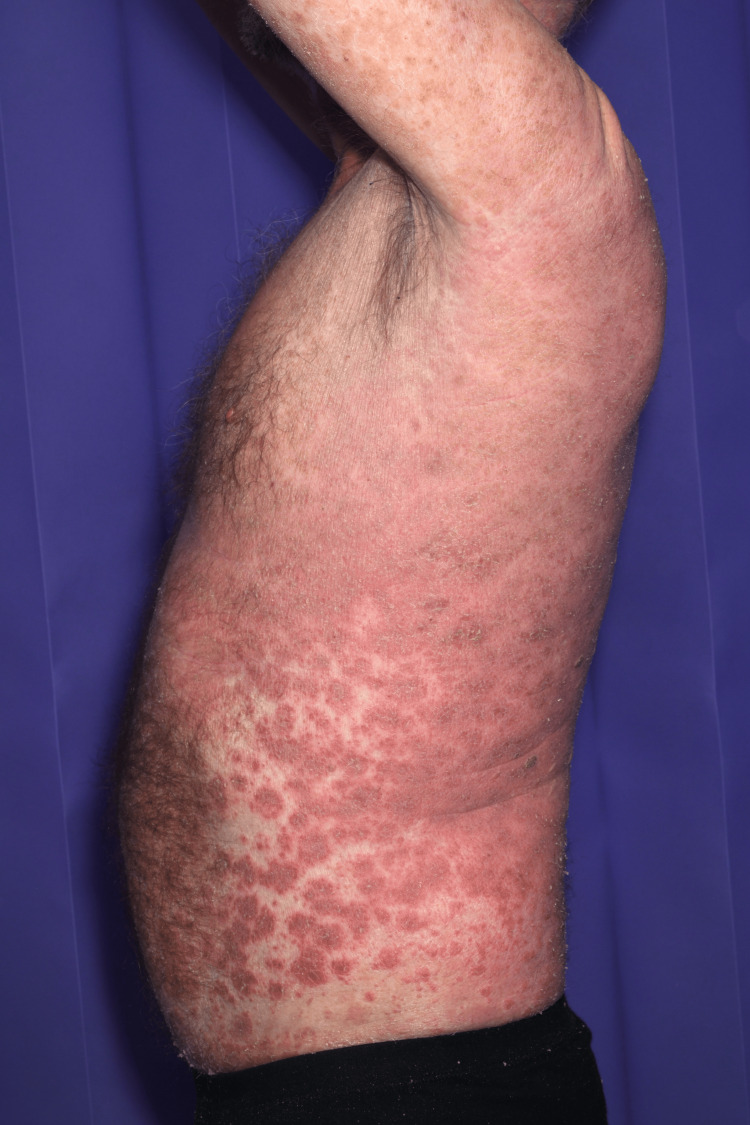
The patient had SJS rashes spread all over his trunk SJS: Steven-Johnson Syndrome.

A 64-year-old male patient, known to have atrial fibrillation (previously on bisoprolol 2.5 mg and rivaroxaban 20 mg), presented to the emergency department (ED) complaining of a generalized rash all over the body two weeks after taking bumetanide (started on 1 mg once a day (OD) dose by his general practitioner (GP) for heart failure. Rashes started on his palm, spreading on his arms, hands (Figure 1), legs (Figure 2), trunk, and whole abdomen (Figure 3), and then to the rest of the body. The patient described rashes as itchy, painful, burning, crusty, and peeling in some areas. Rashes were associated with joint pain and polydipsia. He appeared ill with dehydration, and examination revealed widespread confluent erythema multiforme all over the body with mucosal involvement and eye involvement. Vitals were stable throughout except for tachycardia. Naranjo's criteria score was 7 based on clinical onset and resolution of symptoms.

The clinical presentation in this patient was most consistent with Stevens-Johnson syndrome (SJS) rather than toxic epidermal necrolysis (TEN) or drug reaction with eosinophilia and systemic symptoms (DRESS). The patient developed mucocutaneous lesions characterized by targetoid eruptions and epidermal detachment involving less than 10% of the body surface area, fulfilling the diagnostic criteria for SJS. In contrast, TEN typically demonstrates more extensive epidermal detachment, exceeding 30% of the body surface area, while SJS-TEN overlap involves 10-30%. Additionally, the absence of systemic organ involvement, lymphadenopathy, and significant eosinophilia argued against DRESS. The onset of symptoms occurred approximately two weeks after initiation of bumetanide, the only newly introduced medication, and the condition improved markedly following its withdrawal, further supporting a drug-induced SJS rather than TEN or DRESS.

The white cell counts(18.63 x 10⁹/L) were elevated. Blood tests, including viral and autoimmune screening, were negative. On SCORTEN criteria, it was 3 for SJS. Medical photography was arranged urgently and reviewed by a dermatologist, who clinically diagnosed the condition as SJS. The presumptive cause was bumetinide. A regimen of 60 mg prednisolone once a day for seven days was started with a plan to reduce the dose gradually if rashes get better, along with stomach and bone protectors. Along with this, symptomatic management was started with fucibet cream and hydromol for cracked/crusted lesions on palms and soles, along with anti-histamine tablets for itching. Because of the mucosal involvement of the mouth, he was started on benzydamine mouthwash. Due to eye involvement, he was consulted by an ophthalmologist, and eye care was provided.

Treatment outcome

Over the next two weeks, the patient showed gradual re-epithelialization and mucosal healing. He was discharged home with a reduced dose of prednisolone with a follow-up plan in the clinic and advised to avoid bumetanide and related drugs. Initial presentation was that bumetanide 1 mg was started on 5th April, and the patient presented to us on 22nd April. Bumetanide was withheld on admission, and the patient remained admitted. By May 8th, improvement was observed in skin and mucosal healing.

## Discussion

Stevens-Johnson syndrome (SJS) is a rare, life-threatening mucocutaneous disorder characterised by epidermal necrosis and detachment, typically involving less than 10% of the total body surface area [1]. It lies on a disease spectrum with toxic epidermal necrolysis (TEN), the main distinction being the extent of skin involvement. Mortality rates for SJS range between 10% and 15%, increasing with age, comorbidities, and delayed diagnosis [2]. In most cases, SJS is induced by medications, with antibiotics, antiepileptic drugs, and non-steroidal anti-inflammatory agents being the most frequently implicated [3]. Loop diuretics, including bumetanide, are not widely recognized as common triggers, but isolated reports have described similar hypersensitivity reactions [4,5]. In our case, the patient developed SJS two weeks after initiating bumetanide for heart failure, a timeline consistent with delayed-type drug hypersensitivity reactions.

The immunopathogenesis of SJS involves activation of drug-specific cytotoxic CD8+ T cells and natural killer cells, leading to widespread keratinocyte apoptosis through Fas-FasL interactions, perforin/granzyme release, and granulysin secretion [6]. Genetic predispositions, such as specific human leukocyte antigen (HLA) alleles, are well-established for several drugs (e.g., carbamazepine, allopurinol) but have not been demonstrated for bumetanide [7].

Early diagnosis and immediate discontinuation of the offending agent remain the most crucial steps in management [8]. Supportive care is the cornerstone of therapy, including meticulous wound care, fluid and electrolyte replacement, infection prevention, nutritional support, and pain control. Multidisciplinary management, encompassing dermatology, ophthalmology, and intensive care, is essential to optimize outcomes [9]. Although systemic corticosteroids, intravenous immunoglobulin (IVIG), and cyclosporine have been used in some cases, the evidence regarding their efficacy remains inconclusive [10,11].

This case highlights the need for clinicians to remain alert for severe cutaneous adverse reactions (SCARs) even with medications traditionally considered low risk, such as bumetanide. Vigilant monitoring during the initial weeks of therapy, prompt recognition of prodromal symptoms, and timely withdrawal of the offending drug are key to reducing morbidity and mortality. Furthermore, detailed reporting of such rare cases is crucial to enhance understanding, guide pharmacovigilance, and potentially identify genetic risk factors that predispose certain individuals to SJS.

## Conclusions

This case highlights the importance of recognising bumetanide, a commonly prescribed loop diuretic, as a potential, though rare, cause of SJS. The strong temporal relationship between drug initiation and symptom onset, exclusion of other possible triggers, and clinical improvement following withdrawal support a likely causal association. SJS is a potentially fatal, multisystem disorder most often induced by medications. Clinicians should maintain a high index of suspicion for SJS when patients develop mucocutaneous or systemic symptoms shortly after starting new therapies. Given the multi-organ nature of this condition, early multidisciplinary involvement is essential to optimise outcomes.

While systemic corticosteroids and other immunomodulatory agents may be considered, their efficacy remains uncertain. This case contributes to growing awareness of atypical, drug-induced SJS triggers and underscores the need for continued pharmacovigilance and post-marketing surveillance of bumetanide and other loop diuretics. Ultimately, early recognition, immediate discontinuation of the offending drug, and prompt supportive management remain the cornerstone of effective care.
